# Self-supervised representation learning using feature pyramid siamese networks for colorectal polyp detection

**DOI:** 10.1038/s41598-023-49057-6

**Published:** 2023-12-08

**Authors:** Tianyuan Gan, Ziyi Jin, Liangliang Yu, Xiao Liang, Hong Zhang, Xuesong Ye

**Affiliations:** 1 Biosensor National Special Laboratory, College of Biomedical Engineering and Instrument Science, Zhejiang University, Hangzhou, 310027 China; 2 Department of Gastroenterology, Endoscopy Center, Sir Run Run Shaw Hospital, School of Medicine, Zhejiang University, Hangzhou, 310016 China; 3 Department of General Surgery, Sir Run Run Shaw Hospital, School of Medicine, Zhejiang University, Hangzhou, 310016 China

**Keywords:** Colonoscopy, Biomedical engineering, Colorectal cancer

## Abstract

Colorectal cancer is a leading cause of cancer-related deaths globally. In recent years, the use of convolutional neural networks in computer-aided diagnosis (CAD) has facilitated simpler detection of early lesions like polyps during real-time colonoscopy. However, the majority of existing techniques require a large training dataset annotated by experienced experts. To alleviate the laborious task of image annotation and utilize the vast amounts of readily available unlabeled colonoscopy data to further improve the polyp detection ability, this study proposed a novel self-supervised representation learning method called feature pyramid siamese networks (FPSiam). First, a feature pyramid encoder module was proposed to effectively extract and fuse both local and global feature representations among colonoscopic images, which is important for dense prediction tasks like polyp detection. Next, a self-supervised visual feature representation containing the general feature of colonoscopic images is learned by the siamese networks. Finally, the feature representation will be transferred to the downstream colorectal polyp detection task. A total of 103 videos (861,400 frames), 100 videos (24,789 frames), and 60 videos (15,397 frames) in the LDPolypVideo dataset are used to pre-train, train, and test the performance of the proposed FPSiam and its counterparts, respectively. The experimental results have illustrated that our FPSiam approach obtains the optimal capability, which is better than that of other state-of-the-art self-supervised learning methods and is also higher than the method based on transfer learning by 2.3 mAP and 3.6 mAP for two typical detectors. In conclusion, FPSiam provides a cost-efficient solution for developing colorectal polyp detection systems, especially in conditions where only a small fraction of the dataset is labeled while the majority remains unlabeled. Besides, it also brings fresh perspectives into other endoscopic image analysis tasks.

## Introduction

Colorectal cancer (CRC) stands as a primary cause of cancer-related mortality worldwide, afflicting individuals in both western and eastern countries. To mitigate the incidence and mortality rates of CRC, early-stage lesions such as colorectal polyps must be identified and meticulously removed through colonoscopy, a critical clinical tool for diagnosis^1,2^. Recent advancements in computer-aided techniques employing convolutional neural networks (CNNs) have enabled endoscopists to detect and diagnose colorectal diseases more simply in real-time colonoscopy^3–6^. Nonetheless, the efficacy of these CNN-based approaches is intrinsically linked to the quantity of annotated image data utilized for training. Unfortunately, annotating colonoscopic images proves to be a costly and time-intensive endeavor, necessitating the expertise of proficient clinicians. Therefore, the quantity of annotated medical data available for training is often constrained, in stark contrast to natural images that can be readily annotated via crowdsourcing methods. Two strategies have been proposed to address this issue. The first involves supervised pre-training on a large-scale labeled natural dataset, such as ImageNet, followed by supervised fine-tuning on the target medical dataset with limited labels^7–12^. The second strategy involves self-supervised pre-training on a large-scale unlabeled dataset within the specific medical domain, followed by supervised fine-tuning on the target dataset of the same domain with scarce annotations^13–16^. Although numerous studies have employed the former strategy for disease diagnosis in colonoscopy, the latter strategy, which utilizes self-supervised techniques to learn representations more pertinent to the colonoscopic domain, warrants greater attention. After all, unlabeled image data is abundant and readily available even in the medical field.

Several self-supervised learning (SSL) techniques have demonstrated their efficacy in downstream classification tasks, achieving comparable or even superior results when compared to supervised ImageNet pre-training^17–23^. However, a gap still exists between image-level pre-training that utilizes global features only and target dense prediction tasks, such as polyp detection. It has been shown in^24^ that superior image classification performance does not necessarily guarantee more precise object detection. Therefore, there is a pressing demand for customized SSL approaches that are tailored specifically for the polyp detection task.

In this study, inspired by advanced supervised object detectors that typically predict objects on multi-level fused features^25^, we propose a novel feature pyramid siamese network structure (FPSiam) to perform self-supervised pre-training on a large-scale dataset without the need for human annotations. Subsequently, we fine-tune the detectors end-to-end using only a small amount of labeled data. FPSiam considers the SSL procedure to be a global and local layer-wise instance discrimination rather than just a global one. To this end, we leverage a feature pyramid structure that accepts representations from various layers of the backbone network as input and generates dense fused global and local projections. Unlike other state-of-the-art (SOTA) SSL frameworks, which only output a single global projection vector, our approach naturally preserves both local and global information, a crucial requirement for the polyp detection task. We also introduce a similarity loss function that extends the conventional negative cosine similarity to an additive version. We conduct a comprehensive comparison of our FPSiam pre-trained feature encoder with its ImageNet-supervised pre-trained counterpart, an approach dominant for years, to evaluate their respective transfer abilities in the downstream colorectal polyp detection task. Furthermore, we present the performance of other SOTA SSL algorithms as well as a randomly initialized network as a point of reference.

Our main contributions are summarized as follows:

- To the best of our knowledge, there has been no prior utilization of SSL frameworks in the detection of colorectal polyps. In this study, we undertake a comprehensive investigation of various advanced SSL methods for the task of polyp detection during colonoscopy. Our findings demonstrate the feasibility of establishing competitive colorectal polyp detectors using limited labeled data and abundant unlabeled data. This holds significant potential for both research and clinical applications.
- We introduce a novel SSL method customized for polyp detection tasks, named FPSiam. We propose the feature pyramid encoder module to leverage both global and local feature projections to pre-train highly discriminative representations. Our proposed method outperforms recent SOTA SSL methods as well as the conventional supervised pre-training using ImageNet. By bridging the gap between self-supervised pre-training and dense prediction tasks, FPSiam proves to be a promising solution for the polyp detection task.

## Related works

### Computer-aided polyp detection from colonoscopy

Colorectal cancer is a major cause of cancer-related death worldwide. Early detection and eradication of colorectal polyps is assumed to be an effective approach to reducing the incidence and mortality of CRC. Recently, computer-aided diagnosis systems have become a popular method to assist endoscopists and address human error by indicating the presence and location of polyps during real-time colonoscopy. With the development of deep learning technology, many studies show remarkable performance in automatic colorectal polyp detection. For example, Pacal et al.^5^ employed Scaled-YOLOv4 with different backbones such as CSPNet, ResNet, DarkNet, and Transformer for polyp detection. Karaman et al.^26^ integrated the artificial bee colony algorithm (ABC) into the YOLO baselines to optimize the hyper-parameters and conducted comprehensive studies on Scaled-YOLOv4. Furthermore, they utilized the ABC algorithm to find the optimal activation functions and hyper-parameters for the YOLOv5 detector and successfully obtained much higher performance in real-time polyp detection^27^. Lima et al.^28^ presented a two-stage polyp detection method for colonoscopy images using salient object-extracted maps and transformers. Although the above-mentioned works achieved excellent results, they were all based on supervised learning. Yet, this training paradigm needs large amounts of labeled data, which requires massive effort from colonoscopy experts. Different from the previous studies, we aim to apply the self-supervised learning framework to the colorectal polyp detection task.

### Transfer learning for medical image analysis

Transfer learning from ImageNet pre-trained model is the most common approach used in medical image analysis for different imaging modalities including radiology^7^, histopathology^8^, and endoscopy^9–12^. Although the distribution of natural images and medical images is quite different, multiple studies^29–31^ have proved that this paradigm can improve model performance in various task settings. However, with further detailed investigation, Raghu et al.^32^ found that transfer learning from ImageNet can speed up convergence especially when the labeled data is limited, but it does not always improve the performance in medical image analysis tasks. Liang et al.^33^ indicated that transfer learning benefits from the model pre-trained on in-domain data. However, the procedure of gathering medical image data with annotations from the same domain is time-consuming and expensive. On the contrary, unlabeled medical data is easy to obtain. Therefore, self-supervised learning becomes a feasible candidate for medical image analysis tasks with limited labeled data.

### Self-supervised learning

Self-supervised learning, or unsupervised visual representation learning, aims to obtain good representations from large-scale datasets without annotations and brings benefits to the training procedure of different downstream tasks. Many different handcrafted pretext tasks for self-supervised training have been proposed to learn such representations. Examples including RelativePosition^34^, Jigsaw^35^, Rotation^36^, Colorization^37^, DeepCluster^38^ and BigBiGAN^39^. However, even have been shown to be useful, these methods are being eliminated by contrastive learning (CL).

Contrastive learning is the most cutting-edge type of self-supervised learning framework. The basic idea of contrastive learning is that different transformations of a sample image have similar representations and these representations should be different from the different sample images. The unlabeled data is used to minimize a loss function named contrastive loss to train the backbone network. Currently, some state-of-the-art (SOTA) contrastive learning frameworks such as CPC^40^, MoCo^17,18,41^, SimCLR^19,20^, BYOL^21^, SimSiam^23^ and SwAV^22^ have greatly closed the gap between unsupervised and supervised representation learning or even surpassed the latter in many computer vision tasks. Therefore, self-supervised pre-training has the potential to serve as an alternative to ImageNet-supervised pre-training in several specific applications.

### Self-supervised learning for medical image analysis

In spite of the big success achieved by self-supervised learning in the nature image domain, its application in medical image analysis is still in its infancy. Due to the big difference between the distribution of medical images and natural images, how to effectively apply the existing SSL frameworks to solve medical image analysis tasks has become a research hotspot. While some works have attempted to design domain-specific pretext tasks^42–46^, other works try to exploit improved version of the existing advanced contrastive learning frameworks to medical data^13,14,16,47–53^.

Contrastive predictive coding (CPC) is a contrastive learning framework that can be applied to many different data types and it first proposed InfoNCE loss for contrastive learning. For image data, CPC learns the feature representation of spatial information by predicting the subsequent image blocks using the embeddings encoded from front image blocks. Inspired by CPC, TCPC^13^ was proposed to learn 3D feature representation from the sub-volumes containing the lesion areas and train a neural network for classifying the 3D CT images. MoCo is another SSL framework that utilizes contrastive learning. It increases the number of negative samples by using a momentum-updated queue of previously seen samples. Based on MoCo, Sowrirajan et al.^14^ proposed a MoCo-CXR framework for classification tasks on unlabeled chest X-ray datasets. Different from MoCo, SimCLR chooses to use a larger batch to provide large-scale negative samples. Benefiting from that, Azizi et al.^48^ applied the SimCLR framework to skin image analysis. BYOL is an approach different from its previous ones, it first utilizes only positive samples in contrastive learning. To be specific, BYOL trains an online network on one augmented view of an image to predict the representation of another augmented view of the same image encoded by the target network. Based on BYOL, Xie et al.^16^ proposed the prior-guided local (PGL) framework for 3D medical image segmentation. More detailed information about the studies using self-supervised learning for medical image analysis can be found in Table 1.

According to Table 1, prior works have proved the effectiveness of self-supervised learning in numerous medical imaging modalities, especially the domain of radiology including MRI, CT, and X-ray. However, there is limited work investigating the performance of the SSL paradigm for endoscopic data, which is another important imaging branch for modern medicine. Distinct from other medical imaging data comprised of still images, endoscopic images are frames derived from dynamic video streams, exhibiting pronounced homogeneity between successive frames. This characteristic has the potential to ruin the performance of some vanilla self-supervised learning frameworks that even have been verified to work well in other medical imaging modalities. Furthermore, despite classification and segmentation tasks being widely studied, the exploration of object detection, another important downstream task, in a self-supervised manner gains insufficient scholarly attention. To the best of our knowledge, we are the first to investigate the SSL methodology for the colorectal polyp detection task during colonoscopy video.

**Table 1:** Overview of studies using self-supervised learning for medical image analysis.

Year	Authors	Imaging modality	Clinical domain	Task	SSL method	Dataset	Metric	Performance
2019	Zhuang et al.^42^	MRI	Radiology	Brain tumor segmentation	Rubik’s cube	BraTS-2018	mIoU	0.773
2020	Nguyen et al.^43^	CT	Radiology	Organ-at-risk segmentation	Predict whether the slice is corrupted as well as its index	StructSeg	Dice	0.917
2020	Zhu et al.^13^	CT	Radiology	pulmonary nodules classification	CPC (modified)	LUNA16	Accuracy	0.994
2020	Xie et al.^16^	CT	Radiology	Kidney organ and tumour segmentation	BYOL (modified)	KiTS	Dice	0.843
2021	Ewen et al.^44^	CT	Radiology	COVID-19 classification	Rotation Prediction	SPGC COVID-19 dataset	Accuracy	0.867
2021	Kaku et al.^47^	Fundus image	Ophthalmology	Diabetic retinopathy classification	MoCo (modified)	EyePACS	AUC	0.966
2021	Sowrirajan et al.^14^	Chest X-ray	Radiology	Pleural effusion classification	MoCo (modified)	CheXpert	AUC	0.953
2021	Azizi et al.^48^	Skin image	Dermatology	Skin conditions classification	SimCLR (modified)	Derm	Accuracy	0.700
2021	Zhao et al.^45^	MRI	Radiology	Alzheimer’s disease classification	Autoencoder (modified)	ADNI1	Accuracy	0.870
2022	Manna et al.^46^	MRI	Radiology	ACL tear classification	Jigsaw Puzzle	MRNet	AUC	0.848
2022	Ciga et al.^49^	Whole slide images & Image Patches	Pathology	5 cancer classification tasks	SimCLR	57 public datasets	F1-score	0.779
2022	Benvcevic et al.^50^	Chest X-ray	Radiology	13 different anomalies detection	SimCLR	VinDr-CXR	mAP	0.142
2022	Hossain et al.^51^	Chest X-ray	Radiology	COVID-19 classification	SwAV	COVID-19 radiography database	Accuracy	0.992
2023	Li et al.^52^	OCT	Ophthalmology	Retinal edema segmentation	BYOL	RESC	Dice	0.689
2023	Chhipa et al.^53^	Whole Slide Images	Pathology	Breast cancer classification	SimCLR (modified)	BreakHis	Accuracy	0.888

## Methods

**Figure 1:**
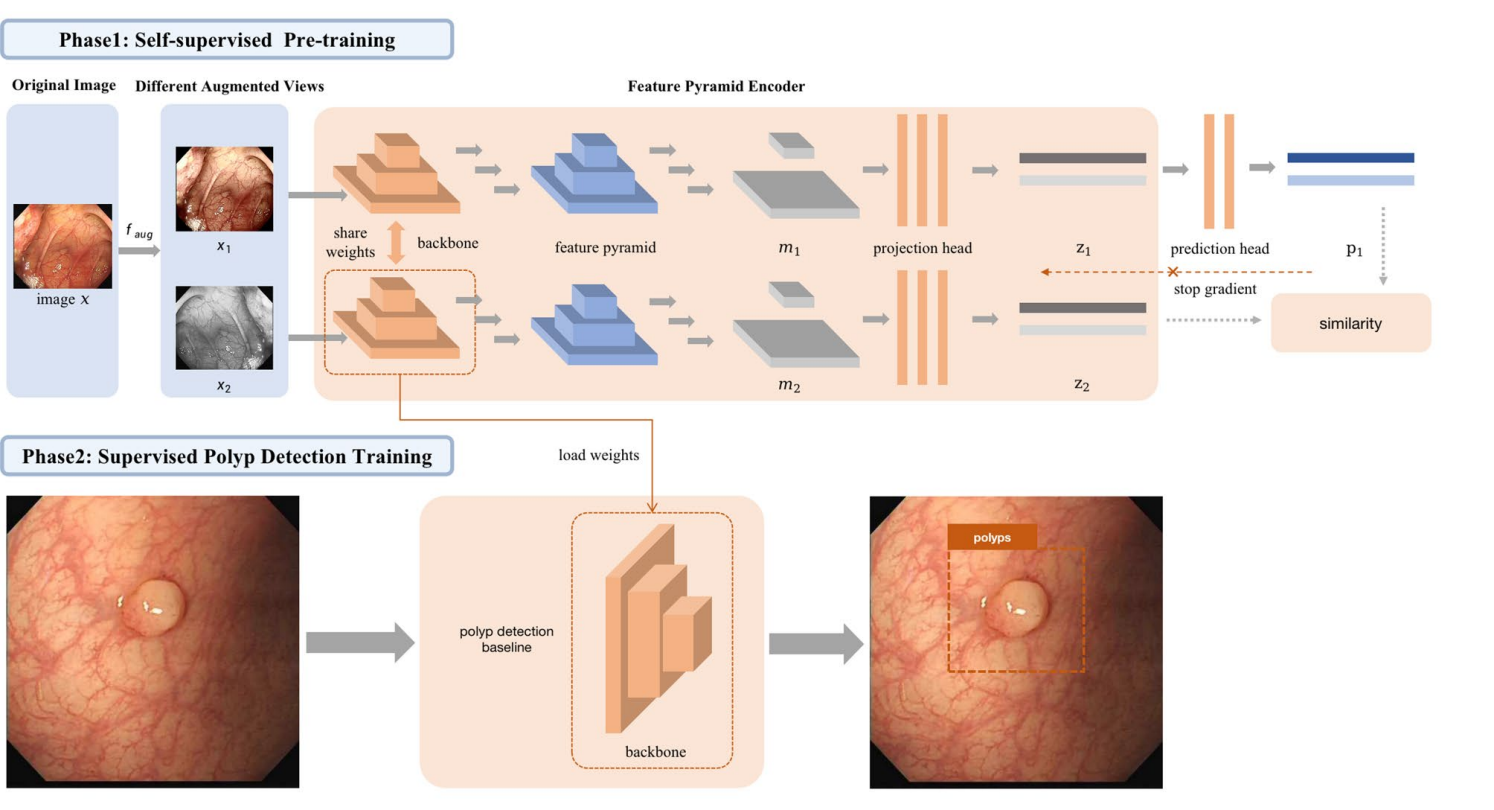
The overall workflow of the proposed self-supervised representation learning framework FPSiam.

In this study, we use the self-supervised learning paradigm to enhance the detection of colorectal polyps. Our algorithmic framework involves a series of steps, as follows. First, we employ the self-supervised learning approach to pre-train the backbone network using unlabelled colonoscopic images. This pre-training phase facilitates the acquisition of robust visual representations in the colonoscopy domain by the network. Subsequently, we perform a supervised end-to-end fine-tuning step using annotated colonoscopic images to accomplish polyp detection in downstream tasks. The whole study workflow is summarized in Fig. 1.

### Datasets

We employ the LDPolypVideo^54^ and CVC-VideoClinicDB^55,56^ datasets in this study. The LDPolypVideo dataset is utilized for both pre-training and downstream fine-tuning stages, while the CVC-VideoClinicDB dataset is exclusively utilized for end-to-end fine-tuning. Our aim in using the latter is to evaluate the transferability of the pre-trained weights to the same task in different in-domain datasets.

#### LDPolypVideo

The LDPolypVideo dataset^54^ was publicly released at MICCAI2021, and to the best of our knowledge, it represents the most extensive publicly available colonoscopy video database. It comprises 40,266 frames of 200 polyps extracted from 160 colonoscopy videos, each with bounding boxes for every polyp. Additionally, it contains 103 videos, comprising 861,400 frames, which have only been simply annotated with video-level annotations that indicate the presence of polyps. The dataset exhibits a broad range of polyp types, sizes, and morphologies, all captured within complex bowel environments, including motion blurs and specular reflections. We utilize the unlabeled video frames to pre-train our self-supervised model and divide the labeled frames into LDPolypVideo$_{train}$ set and LDPolypVideo$_{test}$ set according to the original split in the paper. Finally, the LDPolypVideo$_{pretrain}$ set, LDPolypVideo$_{train}$ set, and LDPolypVideo$_{test}$ set contains a total of 103 videos (861,400 frames), 100 videos (24,789 frames), and 60 videos (15,397 frames), respectively. More detailed information about the dataset can be found in the conference paper^54^.

#### CVC-VideoClinicDB

CVC-VideoClinicDB^55,56^ comprises over 40 short and long video sequences extracted from routine colonoscopy examinations conducted at the Hospital Clinic of Barcelona, Spain. This comprehensive database covers diverse scenarios that a computer-aided polyp detection system may encounter and is tailored for the GIANA challenge of MICCAI. Notably, the polyp frames have been meticulously labeled and reviewed by clinical experts. While only the training data consisting of 18 sequences is available with annotations, we have manually partitioned it into two sets: CVC-VideoClinicDB$_{train}$ (14 video sequences; 9470 images) and CVC-VideoClinicDB$_{test}$ (remaining 4 video sequences, numbered #2, 5, 10, and 18; 2484 images) following^57,58^. Further details about the dataset are available on the GIANA website^59^.

### Self-supervised visual representation learning

We adopt SimSiam^23^ as our baseline to learn visual representation from colonoscopic images without annotations. SimSiam is a recently proposed simple but advanced self-supervised method based on siamese networks. It learns meaningful representations by maximizing the similarity between different augmented views of the same images (positive pairs). SimSiam utilizes only a classic stop-gradient operation to prevent the model’s collapsing solutions. Remarkably, it can achieve competitive performance compared to other cutting-edge self-supervised learning methods (e.g. MoCo^17,18^, SimCLR^19,20^, BYOL^21^) even without negative sample pairs, large batches, and momentum encoders.

Specifically, each image *x* is fed into a probabilistic data augmentation function $f_{aug}(\cdot )$ to create two views $x_{1}$ and $x_{2}$ of the same image. The two views are then encoded respectively by an encoder network $f(\cdot )$ to generate representations $z_{1}=f(x_{1})$ and $z_{2}=f(x_{2})$. The encoder $f(\cdot )$ consists of a backbone and a projection MLP head, sharing weights between the two views. The backbone here can be any convolutional neural network (e.g., ResNet^60^, MobileNet^61^). The representations of one branch are then transformed by a prediction MLP head, denoted as $h(\cdot )$, to match the representations of another view. The two output vectors of the input image *x* are obtained as follows:

1 $$\begin{aligned} p_1&= h(f(x_1)) \end{aligned}$$

2 $$\begin{aligned} z_2&= f(x_2) \end{aligned}$$

Next, negative cosine similarity is used to maximize the similarity between the output:

3 $$\begin{aligned} D(p_1, z_2) = - \frac{p_1}{\left\| p_1\right\| _2} \cdot \frac{z_2}{\left\| z_2\right\| _2} \end{aligned}$$

where $\left\| \cdot \right\| _2$ denotes the l2-norm. To prevent model collapse, a stop-gradient operation is implemented by modifying Eq. (3) as:

4 $$\begin{aligned} D(p_1, stopgrad(z_2)) \end{aligned}$$

This means that $z_2$ is treated as a constant in this term. By reversely feeding the two views $x_1$ and $x_2$, the final training loss is defined as follows:

5 $$\begin{aligned} L = \frac{1}{2}D(p_1, stopgrad(z_2)) + \frac{1}{2}D(p_2, stopgrad(z_1)) \end{aligned}$$

### FPSiam pipeline

Given the dense predictive nature of the polyp detection task, we propose the feature pyramid siamese networks (FPSiam) to extend and generalize the existing SimSiam to a dense paradigm. This new pipeline aims to deepen the model’s understanding of the local representation of the input images. The motivation behind proposing FPSiam arises from the observation that almost all SOTA SSL frameworks only utilize the output features of the last layer of the backbone convolutional neural network for subsequent comparison. However, these deep features reflect the global semantic information of the image (i.e., image-level properties), and due to the small size of the feature map, they lack sufficient geometric information and are therefore not conducive to polyp detection. On the other hand, the output features of shallow layers contain relatively more local geometric information and do not require additional computation costs. Therefore, it is believed that by fusing both deep and shallow CNN features, we can obtain a more robust representation that is suitable for the polyp detection task.

**Figure 2:**
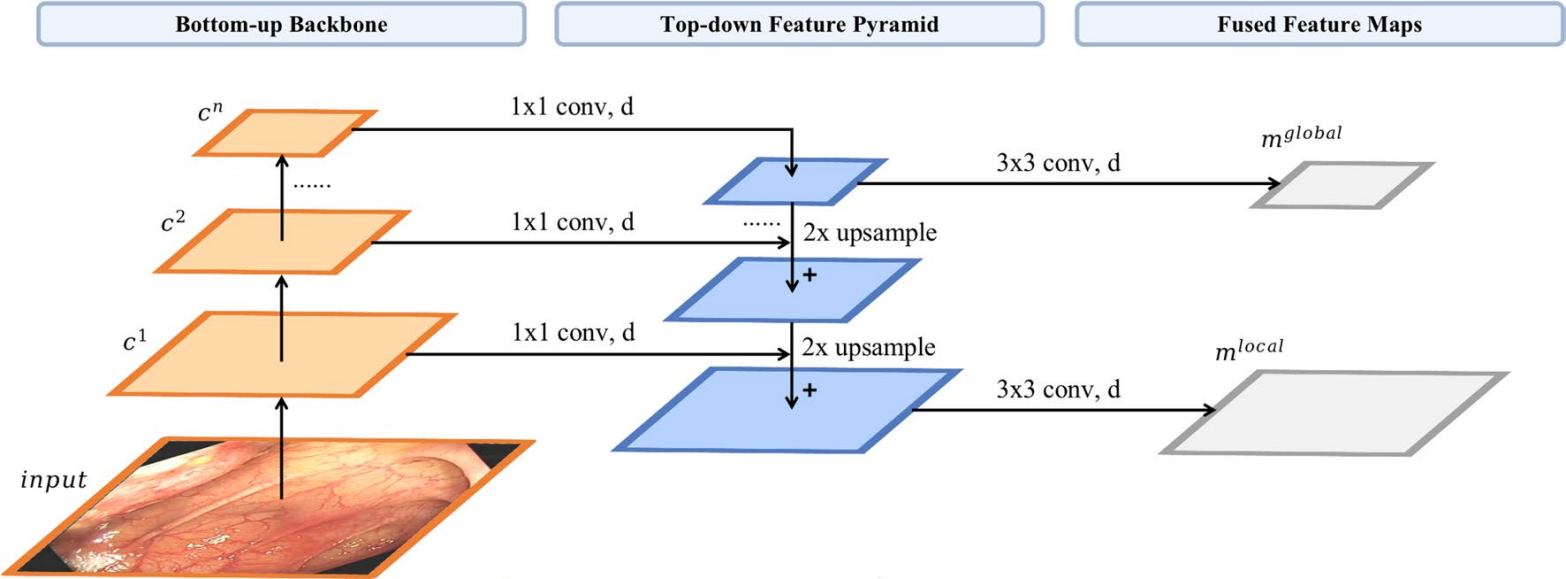
The detailed building components of the feature pyramid.

In practice, compared to the existing paradigm revisited in “Self-supervised visual representation learning”, the core difference lies in the phase between the backbone and the projection head of the encoder. The construction of our feature pyramid involves a bottom-up pathway, a top-down pathway, and lateral connections, as introduced in Fig. 2. Given an input view *x*, the backbone network outputs proportionally sized feature maps at multiple scales with a scaling step of 2, in a fully convolutional fashion. The backbone here can be any convolutional neural network, e.g., ResNet and MobileNet. We denote these feature maps at different levels as $c^i(i=1,2,\ldots ,n)$, $c^n$ represents the feature map output by the latest layer of the backbone. This feed-forward computation procedure of the backbone ConvNet is called the bottom-up pathway. Following that, the top-down pathway generates high-resolution features by upsampling feature maps that are semantically stronger but spatially coarser, extracted from higher pyramid levels. These features are subsequently refined with the aid of the bottom-up pathway’s features through lateral connections, which merge feature maps of equal spatial size from both pathways. Specifically, we employ a low-resolution feature map and increase its spatial resolution by a factor of 2 via nearest-neighbor upsampling for simplicity. The resulting upsampled map is then merged with the corresponding bottom-up map, which has undergone a $1\times 1$ convolutional layer to adjust its channel dimensions, using element-wise addition. This iterative process is repeated until the highest-resolution map is generated. To initiate the iteration, we simply attach a $1\times 1$ convolutional layer to $c^n$ to produce the start feature map. Finally, we apply a $3\times 3$ convolution to the first and last merged maps in order to generate the final fused global and local feature maps, which serve to reduce the aliasing effect of upsampling. These two final fused feature maps are designated as $m^{local}$ and $m^{global}$, corresponding respectively to $c^1$ and $c^n$, which possess the same feature dimensions. The final dense feature maps are then forwarded as input to the shared projection head, which outputs the representation vectors $z^{local}$ and $z^{global}$ for the first and last pyramid levels. The projection head utilized here adheres to the same architecture as the existing projection head in SimSiam, containing a global average pooling layer and an MLP. We designate the aforementioned process as the feature pyramid encoder $f_{pyramid}(\cdot )$. In the same manner, we employ a shared prediction head to handle the representation vectors and generate the prediction vectors $p^{local}$ and $p^{global}$ for ultimate comparison. Finally, the encoder and prediction head are trained end-to-end by optimizing a joint pairwise negative cosine similarity loss at local and global feature levels. The total loss for our FPSiam can be formulated as:

6 $$\begin{aligned} L_{local}&= \frac{1}{2}\left( D\left( p_1^{local}, stopgrad\left( z_2^{local}\right) \right) + D\left( p_2^{local}, stopgrad\left( z_1^{local}\right) \right) \right) \end{aligned}$$

7 $$\begin{aligned} L_{global}&= \frac{1}{2}\left( D\left( p_1^{global}, stopgrad\left( z_2^{global}\right) \right) + D\left( p_2^{global}, stopgrad\left( z_1^{global}\right) \right) \right) \end{aligned}$$

8 $$\begin{aligned} L&= \lambda * L_{local} + (1-\lambda ) * L_{global} \end{aligned}$$

where $\lambda$ acts as the weight to balance the terms of local and global feature pyramid levels, satisfying $\lambda \in \left[ 0,1\right]$. The subscripts of {$z_1,z_2$} and {$p_1,p_2$} represent the reverse feeding operation of the two different augmented views to the two pipelines of the siamese networks.

### Network architecture

**Figure 3:**
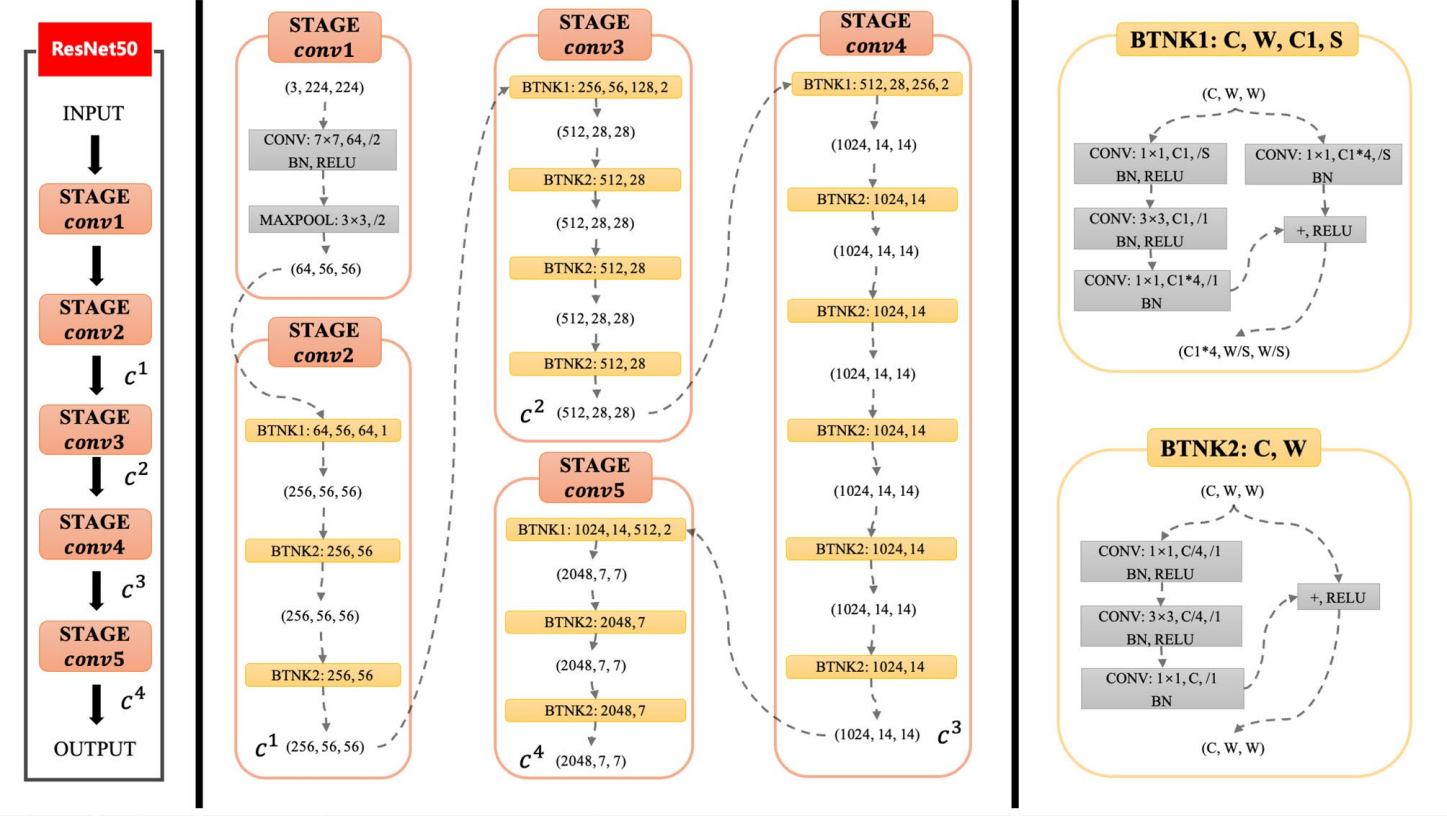
The architecture of the ResNet50 backbone. $c^i(i=1,2,3,4)$ represents the feature maps interacting with the feature pyramid of the proposed FPSiam framework. Each $c^i$ has the same meaning as in Fig. 2.

We use the standard ResNet50^60^ architecture (see Fig. 3) as the backbone network of the encoder for our FPSiam framework. To construct the feature pyramid, we leverage the output of the final layer of each ResNet stage as input feature maps. Although numerous layers of a stage produce feature maps of the same size, we opt for this natural choice as the deepest layer of each stage typically possesses the strongest features. Specifically, we utilize the feature activations of the last residual block of stage {*conv*2, *conv*3, *conv*4, and *conv*5}, denoted as {$c^1, c^2, c^3, c^4$}, which own channel dimensions of {256, 512, 1024, 2048}, correspondingly. We exclude *conv*1 from the pyramid construction due to its excessive memory usage. Because all levels of the pyramid outputs use the shared projection head and prediction head in the FPSiam’s subsequent steps, we fix the feature dimension of the output feature maps to $d = 256$. Therefore, all additional $1\times 1$ and $3\times 3$ convolutional layers within the feature pyramid hold 256-channel outputs. To flatten the output feature maps into feature vectors, we apply a global average pooling layer. Next, a 3-layer MLP projection head (with a hidden layer of 2048 dimensions) is utilized to adjust the feature dimension to 2048. BN is applied to each fully connected (fc) layer, while the output fc layer has BN but no ReLU. The resulting 2048-dimensional output feature vectors are then passed through a 2-layer MLP prediction head with a bottleneck structure. The input and output of this prediction head are also 2048-D, while its hidden layer is 512-D. Only the hidden fc layers have BN applied, while the output fc layer lacks both BN and ReLU. Note that these feature pyramid, projection head, and prediction head are solely used during SSL pre-training and have no influence on the subsequent polyp detection stage.

## Results

### Metrics

The mean average precision (mAP) serves as a metric for evaluating the polyp detection performance of our FPSiam framework and other comparison methods. The calculation of mAP is consistent with the settings of the COCO dataset^62^. Additionally, we report the COCO-style $AP_{50}$ and $AP_{75}$ results, which utilize IoU thresholds of 50% and 75%, respectively. The results are averaged over 5 independent trials.

### Experimental details

We use 4 NVIDIA TESLA A100 40GB GPUs for conducting experiments. Our FPSiam framework is implemented based on *pytorch*^63^ and *mmselfsup*^64^. During SSL pre-training, we set the batch size to 256, which is suitable for typical 4-GPU implementations. The encoder’s backbone is initialized with pre-trained ImageNet weights, which are readily obtainable. To prepare the images for input, they are randomly cropped and resized to $224\times 224$ using a scaling factor between 0.2 and 1.0. For data augmentation, we applied standard techniques, such as color jitter, grayscale, Gaussian blur, and random horizontal flips, as used in SimSiam. Specific settings are kept the same with SimSiam. All fundamental data augmentations are available in *pytorch’s torchvision package*. The feature pyramid weight hyper-parameter related to the FPSiam framework itself is set to 0.5 ($\lambda =0.5$). SGD optimizer with an initial learning rate of 0.05, a momentum of 0.9, and a weight decay of 0.0001 is used for pre-training. The cosine annealing schedule is used to adjust the learning rate according to the current epoch. However, the prediction MLP’s learning rate remained fixed without decaying. We employed batch normalization synchronized across devices, as per SimSiam. The SSL pre-training stage lasts for a total of 200 epochs.

For the downstream polyp detection task, we select two different representative detectors with the same ResNet50 backbone as our baselines. Namely, they are Faster R-CNN and RetinaNet, which are typical two-stage and single-stage detectors, respectively. By utilizing the baseline models that serve as a standard reference point for evaluating other improved models, we can assess the effectiveness of our innovations and improvements. We use their implementations in *mmdetection*^65^ framework. We freeze the layers in the stage *conv1* of the backbone and fine-tune all other layers including BN in an end-to-end manner on the default $1\times$ (12 epochs) schedule settings. The input images are resized to $480\times 480$ during both the training and inference phases. For data augmentation, we only apply random flipping with a probability of 0.5. The initial learning rate of the SGD optimizer is set to 0.002. The other configurations and hyper-parameters are consistent with the default settings in the *mmdetection* framework. Unless explicitly mentioned, all experiments are conducted under the same setup for self-supervised pre-training and downstream evaluation.

### Ablation study

We conducted ablation experiments to investigate the impact of the proposed feature pyramid encoder module, as well as the weight distribution across local and global feature pyramid levels and the strategy of loading an ImageNet pre-trained backbone. The ‘base’ approach, which employs the original encoder, serves as the comparison baseline. We denote the method that replaces the original encoder with the feature pyramid encoder as ‘base+FP’, and the method that initializes the backbone in ‘base’ approach with ImageNet pre-trained weights as ‘base+img-pre’. Through the integration of the feature pyramid encoder and the ImageNet pre-trained backbone initialization with the ‘base’ method, we present our novel approach ‘FPSiam’. To evaluate the feature representation capability of the four aforementioned methods for the downstream polyp detection task, we measure the mAP metric on the LDPolypVideo$_{test}$ dataset. Due to the limitations in computing power, we present solely the outcomes of the Faster R-CNN detector for empirical reference.

The ablation study results are shown in Table 2, where we observe a gradual increasing trend in mAP metric from the ‘base’ to our FPSiam method. Specifically, the detection performance of ‘base+FP’ exhibits an improvement of approximately 2.4 mAP over the ‘base’ method, indicating the efficacy of our proposed feature pyramid encoder. Moreover, we note that ‘base+img-pre’ shows a significant improvement of approximately 8.7 mAP compared to the ‘base’ method, underscoring the vital role played by ImageNet pre-trained weights in extracting superior feature representations for the downstream detection task. In addition, the detection performance of our FPSiam method surpasses that of ‘base+img-pre’ by 1.3 mAP, consolidating the superiority of our approach. We also explore the impact of different hyper-parameter $\lambda$ on the performance of our FPSiam method, with the results summarized in Table 2. We observe a trend where the detection performance initially improves with increasing $\lambda$, before declining beyond a certain threshold. Our FPSiam method obtains the optimal mAP when $\lambda =0.5$, which demonstrates that the balance of global term and local term in loss function (Eq. 8) is significant for our FPSiam method to work. Therefore, we use this as our default setting in other experiments. Although we did not conduct a more fine-grained ablation study on $\lambda$ due to the computational limitations, we found that for the extreme cases of $\lambda =0.0$ and $\lambda =1.0$, our method exhibits better detection performance when $\lambda =1.0$ (+ 0.8 mAP). It is in line with our intuition that local features hold greater relevance for dense prediction tasks like polyp detection, to some extent.

**Table 2:** Empirical ablation study results on LDPolypVideo dataset.

Method	Weight $\lambda$	mAP	AP$_{50}$	AP$_{75}$
Base	N/A	14.3	35.4	6.1
Base+FP	0.5	16.7 (+ 2.4)	42.5	5.9
Base+img-pre	N/A	23.0 (+ 8.7)	47.3	19.6
FPSiam (ours)	0.0	22.1 (+ 7.8)	46.7	17.5
	0.25	23.2 (+ 8.9)	48.0	19.3
	0.5	**24.3** (+ 10.0)	49.6	20.5
	0.75	23.5 (+ 9.2)	47.7	19.8
	1.0	22.9 (+ 8.6)	46.0	20.0

Significant values are in [bold].

### Comparison with traditional transfer learning method

To investigate the benefit of our FPSiam approach in clinical practice, we performed a comparative analysis with the transfer learning (TL)-based polyp detection method, which is presently prevalent in both research and real-world settings. The hyper-parameters and other detailed architecture of the TL model remained consistent with those described in “Experimental details”. Additionally, we included results obtained from a randomly initialized backbone counterpart as a reference. The evaluation was conducted on the LDPolypVideo dataset using COCO-style mAP metrics, as presented in Table 3. Table 3 illustrates that our FPSiam method has achieved noticeably higher detection performance than the comparison TL method for both two-stage Faster R-CNN and single-stage RetinaNet detectors, + 2.3 mAP and + 3.6 mAP, respectively.

**Figure 4:**
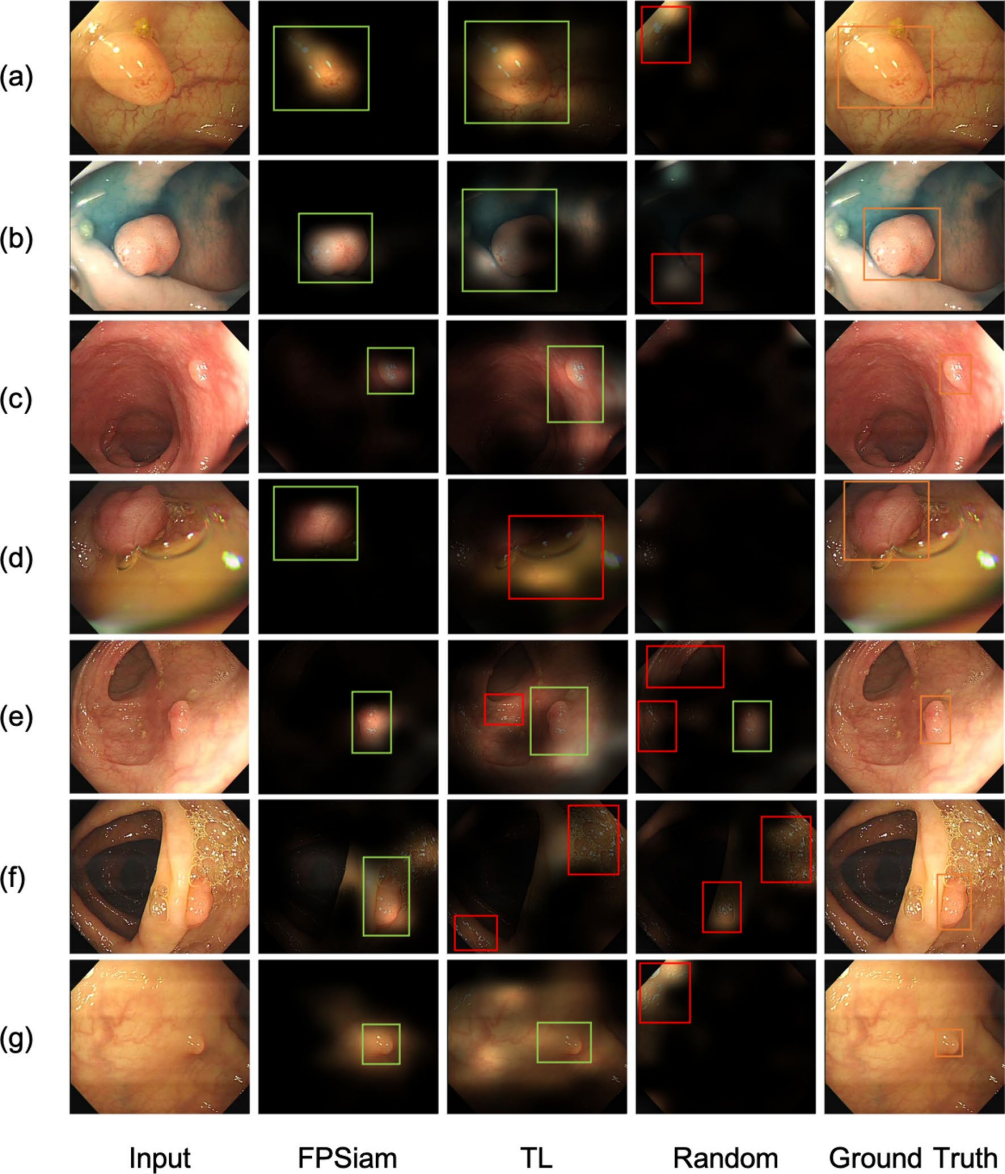
Visualizing the features of seven polyp images using ResNet50 with different weights through Grad-CAM. The regions with higher transparency in the images indicate that the backbone network has paid more attention. The orange bounding boxes represent the ground truth delineating the locations of polyps in each image. The green and red bounding boxes denote accurate and erroneous predictions generated by different methods for each image respectively. FPSiam can activate more accurate polyp regions in the attention maps to make precise bounding box predictions. (**a**–**c**,**e**,**g**) A more precise bounding box localization capability of the FPSiam method than the TL method. TL method fails to locate polyp regions in some cases: (**d**) existence of yellow intestinal fluid, big bubble, and reflection; (**e**) existence of folded intestinal walls; (**f**) existence of folded intestinal walls and dense foam.

In order to provide further insights into the superior polyp detection performance of our FPSiam method, we conducted a visualization analysis of the embedding features outputted by the final layer of the residual network backbone of each approach, utilizing Grad-CAM. As shown in Fig. 4, the FPSiam method is able to more precisely locate the boundaries of polyps and confidently pay less attention to the irrelevant parts of the images. Although the TL method can also notice the location of polyps in most cases, its attention scope is very rough and general. It can even fail in some situations, such as poor bowel preparation (fluid and foam present), poor imaging (reflection), or confusing shapes (existence of folded intestinal walls). The results reflect from the side the compelling localization ability of our FPSiam method to accurately activate polyp regions in the generated attention maps, thus obtaining better polyp detection performance than the TL method.

**Table 3:** Comparisons of the detection performance on LDPolypVideo dataset (ResNet50).

Baseline	Method	mAP	AP$_{50}$	AP$_{75}$
Faster R-CNN	Random init	3.0	9.4	0.9
	TL	22.0	50.0	15.9
	SimSiam	23.0 (+ 1.0)	50.4	17.9
	SimCLR	20.6 (− 1.4)	44.3	16.3
	DenseCL	21.6 (− 0.4)	49.9	14.2
	MoCo	21.5 (− 0.5)	46.9	16.4
	SwAV	23.5 (+ 1.5)	53.9	16.0
	BYOL	23.1 (+ 1.1)	52.1	16.2
	FPSiam (ours)	**24.3** (+ 2.3)	49.6	20.5
RetinaNet	Random init	6.7	17.7	3.6
	TL	21.2	49.1	15.1
	SimSiam	23.4 (+ 2.2)	50.0	18.0
	SimCLR	20.5 (− 0.7)	44.7	16.5
	DenseCL	20.9 (− 0.3)	46.4	15.4
	MoCo	20.8 (− 0.4)	49.5	13.4
	SwAV	23.9 (+ 2.7)	49.9	19.5
	BYOL	23.6 (+ 2.4)	52.4	17.5
	FPSiam (ours)	**24.8** (+ 3.6)	50.4	20.6

Significant values are in [bold].

### Comparison with other state-of-the-art SSL methods

We compared our FPSiam approach with six other state-of-the-art self-supervised learning methods that use CNNs as their backbone. These methods, namely MoCo^17,18^, SimCLR^19,20^, BYOL^21^, SimSiam^23^, SwAV^22^, and DenseCL^66^, were evaluated using ResNet50, pre-trained on the ImageNet dataset, to extract feature representations. For a fair comparison, we applied the default settings of batch size 256 in *mmselfsup* framework for all methods. Table 3 presents the comprehensive results of polyp detection on the LDPolypVideo$_{test}$ dataset.

**Figure 5:**
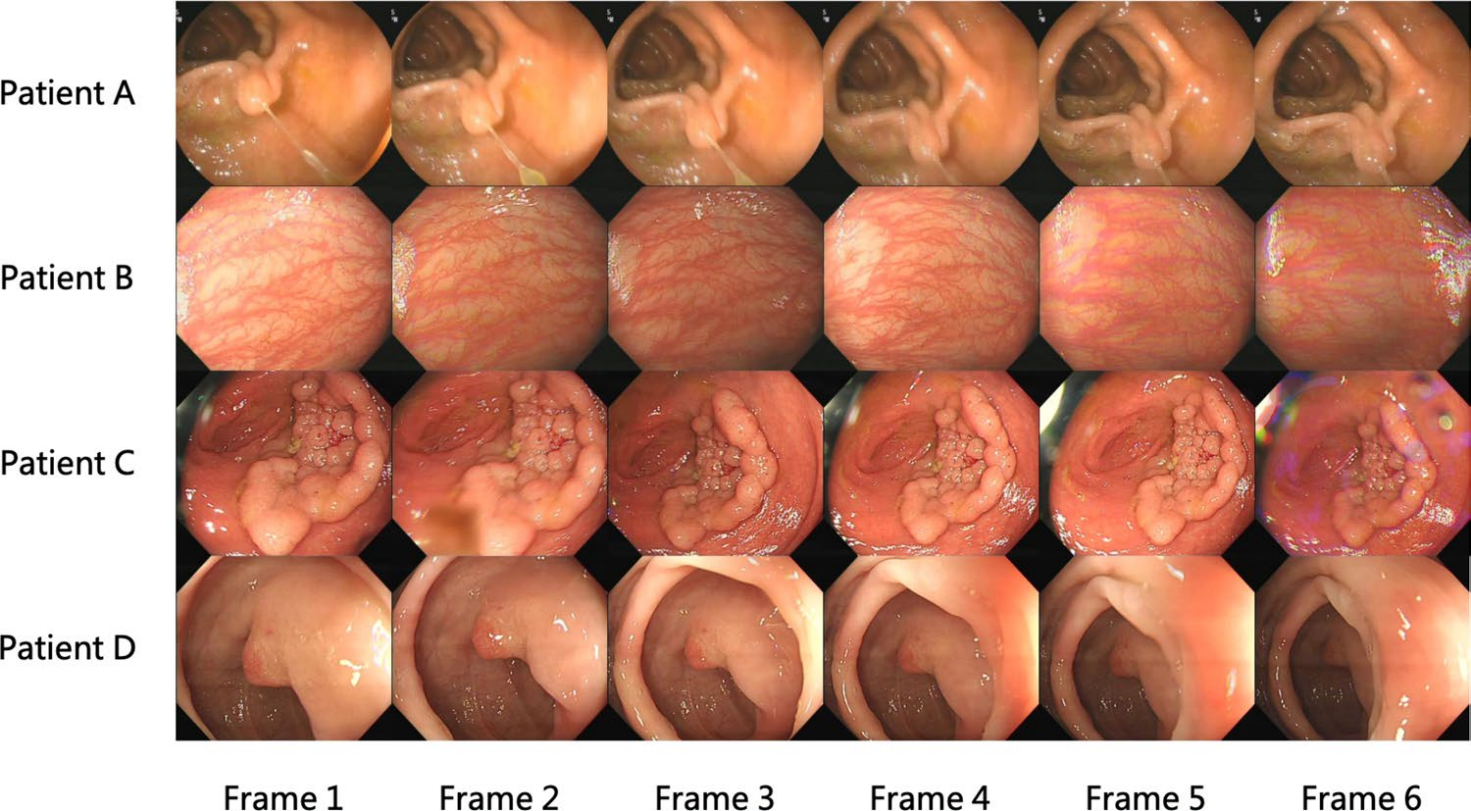
Views of temporally adjacent frames for four patients in LDPolypVideo. For each patient, six frames show a high degree of similarity.

As shown in Table 3, our FPSiam approach outperformed all other advanced SSL methods. When using SimSiam as the benchmark, which also serves as the baseline for our FPSiam method as discussed in “Self-supervised visual representation learning”, we observe a marked decline in the performance of SimCLR, MoCo, and DenseCL. Conversely, SwAV, BYOL, and our FPSiam demonstrate a notable performance improvement. The potential reason is that SimCLR, MoCo, and DenseCL, need both positive and negative pairs in the contrastive manner. However, the LDPolypVideo$_{pretrain}$ dataset used for pre-training is a video-based colonoscopy dataset that has not undergone any frame sampling or other similar operations. As a result, it contains many continuous frames that are temporally adjacent to each other and exhibit high levels of similarity (see Fig. 5). According to common logic, these frames should be considered positive pairs during contrastive learning. But actually, during the SSL pre-training, these frames are treated as negative pairs, thereby confusing the feature encoders.

In addition, among the three methods of MoCo, SimCLR, and DenseCL, SimCLR has the poorest performance, with a decrease of 2.4 mAP and 2.9 mAP in Faster RCNN and RetinaNet detectors compared to the SimSiam baseline, respectively. This may be because, for a fair comparison, we set the batch size for pre-training of all SSL methods to 256. However, SimCLR heavily relies on a large batch size (such as 2048, and 4096) to provide sufficient negative pairs for achieving good performance. As the batch size decreases, its performance deteriorates sharply^21^. Furthermore, among these six SSL methods, DenseCL shows a relatively small performance drop (− 1.4 mAP and − 2.5 mAP compared to the SimSiam baseline, respectively), while our FPSiam method achieves the best performance (+ 3.7 mAP and + 4.3 mAP compared to the worst, respectively). This indicates that learning local feature representations of images during the pre-training stage is crucial for downstream dense prediction tasks like polyp detection.

### Evaluation on the other public dataset

To further assess the transfer ability of the self-supervised feature representation acquired through our FPSiam framework, we transferred the pre-trained weights from the LDPolypVideo$_{pretrain}$ dataset to the polyp detection task of the CVC-VideoClinicDB dataset. We evaluated the detection performance of each comparison method mentioned above, including both state-of-the-art SSL methods and transfer learning method. The comprehensive results are shown in Table 4. Based on the experimental results, we have observed that our FPSiam method achieves the optimal detection performance. Compared with other advanced SSL methods, FPSiam obtains a slightly higher mAP value than the suboptimal BYOL method (+ 2.8 mAP and + 1.1 mAP, respectively), and significantly outperforms both other self-supervised methods (+ 14.4 mAP and + 13.2 mAP compared to the worst SimCLR, respectively) and the widely used transfer learning method (+ 5.3 mAP and + 3.2 mAP, respectively). This finding demonstrates that FPSiam has indeed learned the general feature embeddings of colonoscopic images. It has advantages over the TL method in generalizing to other datasets within the same colonoscopic domain.

**Figure 6:**
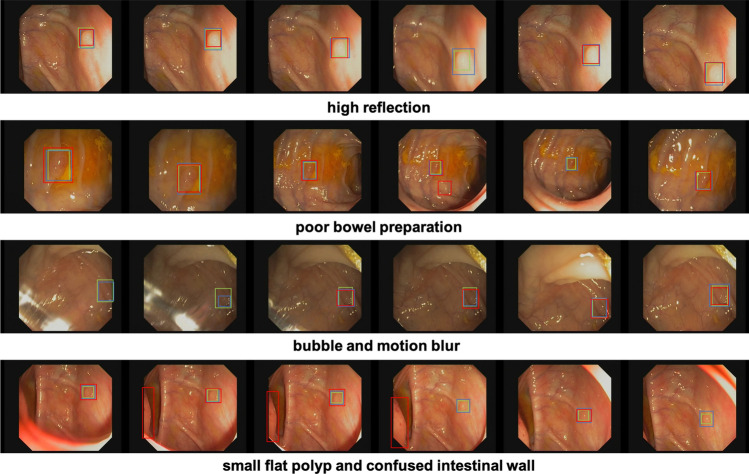
Generalizability of the FPSiam method to another dataset under various challenging conditions. Four representative hard cases in the CVC-VideoClinicDB dataset are used to evaluate FPSiam’s robustness and adaptability. The green bounding boxes are the ground truth of polyps in the frames. Blue bounding boxes and red bounding boxes are the predictions of the FPSiam method and TL method, respectively.

In order to demonstrate the generalization of the FPSiam method to another dataset more intuitively, especially in challenging scenarios such as poor bowel preparation or poor imaging quality, we have visualized its polyp detection results on the CVC-VideoClinicDB dataset. Besides, we also provided the detection results of the TL counterpart for comparison. As shown in Fig. 6, our FPSiam approach illustrates heightened robustness and adaptability to various hard cases, which have to be solved before real-world application, compared to the TL methodology. While the TL method exhibits occurrences of both missed and false detection of polyps, our FPSiam approach consistently obtains precise localization of polyp positions.

**Table 4:** Comparisons of the detection performance on CVC-VideoClinicDB dataset (ResNet50).

Baseline	Method	mAP	AP$_{50}$	AP$_{75}$
Faster R-CNN	Random init	13.4	33.5	5.8
	TL	34.8	70.8	26.6
	SimSiam	33.0 (− 1.8)	75.1	21.2
	SimCLR	25.7 (− 9.1)	62.6	12.4
	DenseCL	29.5 (− 5.3)	67.9	14.1
	MoCo	26.2 (− 8.6)	65.8	12.3
	SwAV	36.5 (+ 1.7)	76.4	26.5
	BYOL	37.3 (+ 2.5)	77.5	27.2
	FPSiam (ours)	**40.1** (+ 5.3)	79.6	32.4
RetinaNet	Random init	16.9	44.3	5.7
	TL	35.0	77.1	25.8
	SimSiam	29.2 (− 5.8)	65.5	19.4
	SimCLR	25.0 (− 10.0)	66.4	9.4
	DenseCL	30.9 (− 4.1)	69.6	23.5
	MoCo	28.5 (− 6.5)	69.8	18.3
	SwAV	36.2 (+ 1.2)	77.9	29.4
	BYOL	37.1 (+ 2.1)	73.7	30.0
	FPSiam (ours)	**38.2** (+ 3.2)	76.3	30.7

Significant values are in [bold].

### Evaluation on the other backbone network

As it is well known that real-time polyp detection is crucial, a swifter backbone network is often used to extract features so as to speed up the detector’s inference procedure in clinical practice. In order to verify the adaptability of our proposed FPSiam method to lightweight backbone networks, we conducted experiments using the renowned MobileNet^61^ architecture. The assessment of detection performance on the LDPolypVideo dataset encompassed all SSL methods mentioned earlier, along with the TL method. The comprehensive outcomes are detailed in Table 5. Table 5 illustrates that our FPSiam approach consistently obtains superior detection performance. It surpasses the vanilla ImageNet transfer learning method significantly (+ 2.7 mAP and + 3.7 mAP, respectively) and outperforms the suboptimal SimSiam approach (+ 1.1 mAP and + 1.3 mAP, respectively). This demonstrates that FPSiam serves not only as a means for acquiring representative knowledge for residual networks but also exhibits adept elasticity to other lighter and faster convolutional neural networks. It stands as a general visual pre-training framework suitable for object detectors’ feature encoders.

**Table 5:** Comparisons of the detection performance on LDPolypVideo dataset (MobileNetV2).

Baseline	Method	mAP	AP$_{50}$	AP$_{75}$
Faster R-CNN	Random init	2.8	9.2	0.9
	TL	19.4	49.5	13.2
	SimSiam	21.0 (+ 1.6)	51.3	13.5
	SimCLR	17.5 (− 1.9)	40.5	14.4
	DenseCL	18.7 (− 0.7)	45.2	13.9
	MoCo	18.4 (− 1.0)	43.5	13.7
	SwAV	20.6 (+ 1.2)	50.8	14.0
	BYOL	20.4 (+ 1.0)	49.9	14.3
	FPSiam (ours)	**22.1** (+ 2.7)	49.5	17.8
RetinaNet	Random init	4.3	10.2	2.3
	TL	18.8	47.8	12.9
	SimSiam	21.2 (+ 2.4)	51.4	13.8
	SimCLR	17.8 (− 1.0)	40.9	14.3
	DenseCL	18.5 (− 0.3)	43.3	13.9
	MoCo	18.3 (− 0.5)	44.5	13.3
	SwAV	20.9 (+ 2.1)	50.0	15.7
	BYOL	20.5 (+ 1.7)	49.7	14.7
	FPSiam (ours)	**22.5** (+ 3.7)	51.2	17.6

Significant values are in [bold].

## Discussion

### Novelty of the proposed FPSiam framework

In this study, we propose FPSiam, an innovative self-supervised learning approach that utilizes data without annotations from medical experts to train the feature encoder. The self-supervised encoder captures general feature representations of colonoscopic images, leading to enhanced performance in the downstream polyp detection task, especially on sparsely labeled datasets. Most contrastive learning methods optimize the encoder network by bringing the representations of positive pairs (different augmentation views of the same images) closer while pushing apart the representations of negative pairs (augmentation views from different images)^17–20,66^. However, unlike natural datasets, original non-curated colonoscopy datasets typically consist of video formats in clinical practice. As depicted in Fig. 5, these datasets contain temporally continuous frames and frames from different views of the same polyp lesion, exhibiting a high degree of similarity. The definition of positive and negative pairs in regular contrastive learning methods would consider these images as negative pairs, causing them to be pushed apart. This approach is clearly unreasonable, as it would confuse the feature encoder network and hinder the learning of better general feature representations for colonoscopic images. Experimental results presented in Tables 3 and 4 confirm this perspective, demonstrating the limitations of MoCo, SimCLR, and DenseCL. Taking into account the unique characteristics of colonoscopic datasets, we have designed FPSiam based on SimSiam, which does not rely on negative pairs.

Additionally, unlike conventional contrastive learning methods, we introduce the feature pyramid encoder module, a crucial component of FPSiam, to effectively extract and fuse feature representations among colonoscopic images from both local and global perspectives. Our ablation results, as presented in Table 2, reveal that for dense prediction tasks like polyp detection, the ability of the backbone network to capture local features is of greater significance compared to its capability to encode global features, to a certain extent. To summarize, the experimental outcomes in “Results” demonstrate the superior detection capability of FPSiam on small labeled colonoscopic polyp datasets compared to other state-of-the-art self-supervised learning methods and transfer learning-based detection methods. Furthermore, the pretext representation learned by FPSiam exhibits enhanced transferability to other similar datasets within the same domain.

### Limitations and future research directions

**Figure 7:**
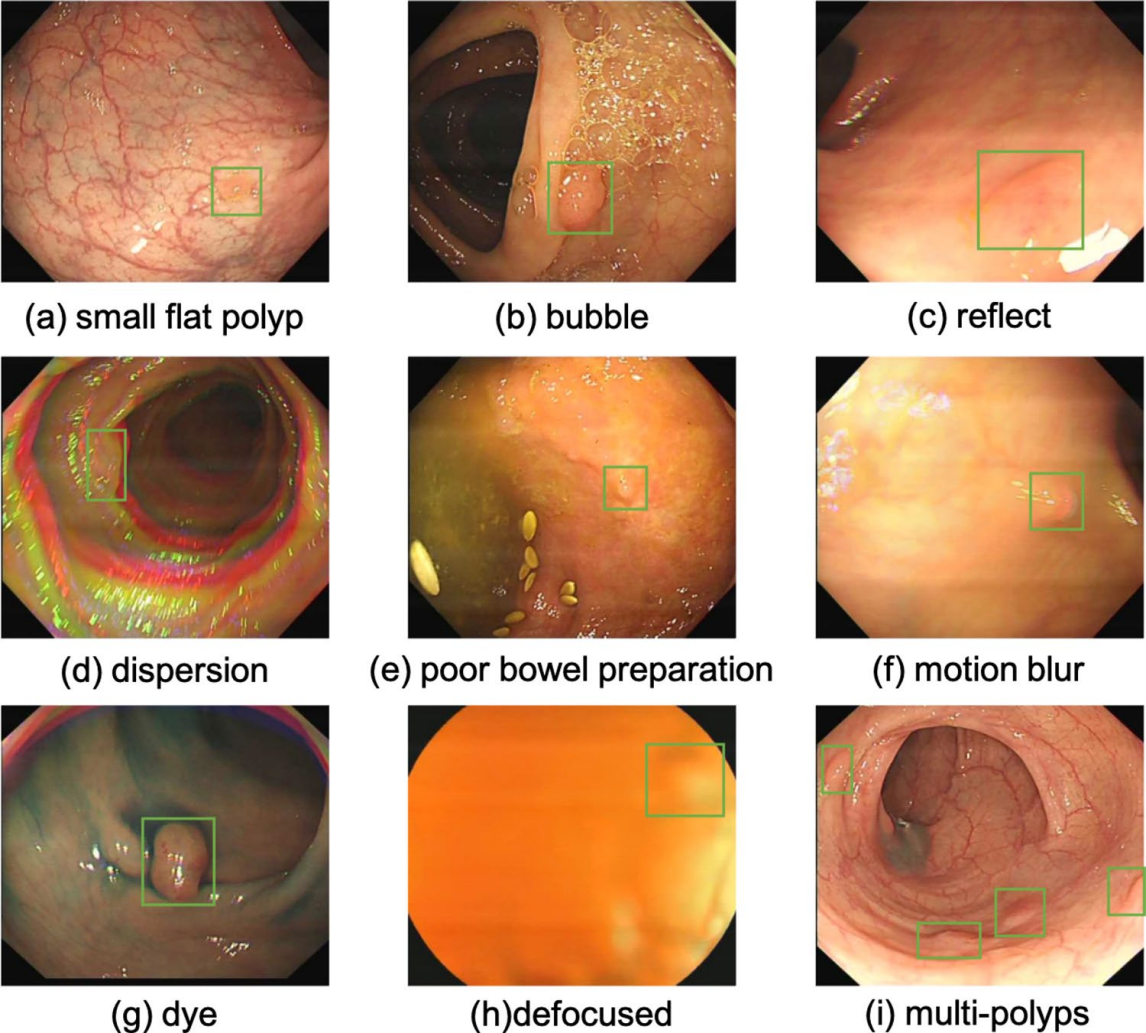
Several representative hard cases in the LDPolypVideo dataset. The green bounding boxes are polyps in the frame, which are difficult to identify due to the complex situations presented in the colonoscopy live video.

This study still has several limitations that need to be addressed. Firstly, the algorithm’s performance requires improvement to bridge the gap for real-world clinical applications. Despite extensive efforts in hyper-parameter optimization through grid search, the achieved detection performance in LDPolypVideo datasets is still approximately from 20.0 to 25.0 mAP. Unlike other public colonoscopy datasets that are often highly curated and balanced, LDPolypVideo represents a more realistic clinical scenario. As depicted in Fig. 7, LDPolypVideo contains challenging situations that are prone to algorithmic failures^67^. These challenging cases encountered in real-world settings result in a significant drop in detection performance, posing problems that must be addressed in the development of colonoscopy CAD systems. Introducing temporal-based techniques may be a potential avenue to address these challenges in video format data. Additionally, with transformer-based large language models (LLMs) gaining prominence in the NLP field and showcasing remarkable generalization capabilities^68–71^, exploring self-supervised methods based on vision transformers (ViTs) such as MoCov3^41^, BEiT^72^, MAE^73^, SimMIM^74^ and MaskFeat^75^ or prompt fine-tuning from large vision models (LVMs), such as the recent SAM model^76^, holds promise for further research directions. Nevertheless, due to the difference in image perception approaches between CNNs and ViTs, employing self-supervised learning for pre-training ViT backbones necessitates greater heterogeneity in data distribution, with the magnitude of data quantity being notably higher compared to CNN^77^. Some SOTA methodologies even demand the utilization of multi-modal data^78–84^. Presently, there is an absence of publicly available datasets meeting the criteria within the domain of colonoscopy. Despite LDpolypVideo standing as a huge dataset, its data homogeneity poses a significant challenge, resulting in a diminished pool of unique instances after deduplication. Therefore, it is imperative to collect a large-scale multi-modal colonoscopy dataset that includes not only colonoscopy videos but also examination reports in the future.

Secondly, it is widely recognized that real-time polyp detection is of paramount importance in clinical practice. In order to provide timely visual feedback to endoscopists and ensure precise surgical manipulation during colonoscopy, computer-aided polyp detection algorithms must achieve real-time performance and low end-to-end latency. Although this study conducted relevant explorations on the faster MobileNet backbone, computational power limits the investigations of a wider range of lightweight backbone networks. Besides, verification of algorithm inference performance on different hardware devices and further validation of other well-known real-time detectors such as the YOLO series^85–90^ constitute essential aspects for future research endeavors.

Finally, in this study, our algorithm focused solely on single-class detection. To validate its scalability, future work should include additional disease types observed during colonoscopy, as well as subtypes of polyps, such as adenomatous and hyperplastic polyps. Thereby promoting the comprehensive application of computer-aided diagnosis systems in colonoscopy.

## Conclusions

In summary, FPSiam exhibits significant performance advancements in polyp detection during colonoscopy, surpassing traditional transfer learning methods and other state-of-the-art self-supervised learning techniques. With its SSL pre-training framework and reduced dependence on annotated data, FPSiam offers a labor-saving and cost-efficient approach to developing colonoscopy CAD systems. Moreover, the proposed FPSiam methodology holds promise for addressing other video-based endoscopic image analysis challenges, particularly in scenarios where only a small portion of the dataset is labeled, and the majority of data remains unlabeled.

### Supplementary Information

Supplementary Information.

## Data Availability

The first dataset (LDPolypVideo) used for the analysis of this article is available in the official github repository of its paper (https://github.com/dashishi/LDPolypVideo-Benchmark). The second dataset used during the analysis is available in the GIANA challenge website (https://giana.grand-challenge.org/).

## Code availability

The code of the FPSiam method is available at https://github.com/GTYuantt/fpsiam.
