# Supplementary material for: Self-supervised representation learning using feature pyramid siamese networks for colorectal polyp detection
Source: Sci Rep. 2023 Dec 8;13:21655. doi: 10.1038/s41598-023-49057-6 (PMC10709402; doi:10.1038/s41598-023-49057-6)
Supplement: Supplementary file 1 — Supplementary Information. [file 41598_2023_49057_MOESM1_ESM.pdf]

Supplementary Information

Self-supervised representation learning using feature pyramid siamese networks for colorectal polyp detection

Tianyuan Gan, Ziyi Jin, Liangliang Yu, Xiao Liang, Hong Zhang, and Xuesong Ye*

Supplementary Note

Supplementary Note 1: Impact of feature pyramid layers for contrastive learning

To demonstrate the effects of feature pyramid layers used for conducting contrastive learning in the proposed FPSiam framework, we performed extra ablation experiments that employed not only the feature maps of the lowest and highest resolutions. The results in Table 2 show that a balanced weight λ for computing the joint pairwise similarity loss of the FPSiam method achieves the best performance. Therefore, for the cases of multiple feature pyramid layers, we formulated the final training loss defined in Section 3.3 as:

$$L_i = \frac{1}{2}(D(p_1^i, stopgrad(z_2^i)) + D(p_2^i, stopgrad(z_1^i)))$$

$$L = \frac{1}{n}\sum_{i \in S} L_i$$

where L_i means the similarity loss of ith feature pyramid layer. The S represents the set of feature pyramid layers used for contrastive learning. The n denotes the number of layers in set S, satisfying n=|S|.

The results in **Supplementary Table 1** illustrate that the introduction of additional mid-level feature pyramids for prediction and loss computation to pretrain the backbone network slightly enhances the performance of downstream polyp detection tasks. However, the marginal improvement suggests that the feature maps at the top and bottom layers are sufficient for robust global and local feature representation. The primary purpose of this study is to validate that the integration of global and local features through the feature pyramid structure for self-supervised pretraining can lead to enhanced performance in downstream polyp detection tasks. Therefore, introducing more feature layers appears unnecessary due to the increased

GPU memory usage and prolonged training time. Given these reasons, subsequent experiments only utilize features of the lowest and highest resolutions for contrastive learning in the manuscript.

Supplementary Note 2: Exploration on local patch-based losses for contrastive learning

For the highest-resolution local feature map m^{local} generated by the feature pyramid mentioned in Section 3.3, directly exploiting a global average pooling operation on it may lead to losing some detailed and geometric information, which is very important for polyp detection. To avoid this problem, we have proposed a modified feature pyramid as shown in **Supplementary Figure 1** to produce a single global feature map and $S_h x S_w$ dense local feature patches for contrastive learning. Each local feature patch passes through the subsequent global average pooling layer alone to preserve local detailed information. The dense version of the final training loss can be formulated as:

$$L_{local(i,j)} = \frac{1}{2}(D(p_1^{local(i,j)}, stopgrad(z_2^{local(i,j)})) + D(p_2^{local(i,j)}, stopgrad(z_1^{local(i,j)})))$$

$$L_{local} = \sum_{i=1}^{S_h} \sum_{j=1}^{S_w} L_{local(i,j)}$$

$$L_{global} = \frac{1}{2}(D(p_1^{global}, stopgrad(z_2^{global})) + D(p_2^{global}, stopgrad(z_1^{global})))$$

$$L = \lambda * L_{local} + (1-\lambda) * L_{global}$$

where $\mathrm{L}_{\mathrm{local(i,j)}}$ represents the similarity loss of the local feature patch in the ith row and j^{th} column of the original local feature map m^{local}. The weight λ is set to 0.5 here according to the results of the ablation experiments in Section 4.3.

As shown in **Supplementary Table 2**, direct global average pooling on the local feature map with the highest resolution during SSL pre-training has little impact on the performance of downstream polyp detection tasks. One possible explanation is that during the self-supervised pre-training stage, the feature encoder can implicitly learn local feature representations in a channel-wise manner from the highest-resolution feature map even with global pooling. As we only transfer the weights of the backbone network in the end-to-end training stage for downstream tasks, the weights of the feature pyramid network (FPN) from the pretraining phase

are discarded. Thus, during SSL pre-training, the backbone network only needs to learn a feature representation suitable for initializing the FPN network, which is sufficient to enhance the performance of the downstream polyp detection task.

Supplementary Table

L_1	L_2	L_3	L_4	mAP	AP_{50}	AP_{75}
✓			✓	24.3	49.6	20.5
✓	✓		✓	**24.5**	49.9	20.4
✓		✓	✓	23.6	50.2	19.3
✓	✓	✓	✓	24.0	49.1	20.2

Supplementary Table 1. Effect of feature pyramid layers for contrastive learning on downstream polyp detection performance. The ablation studies are conducted on the LDPolypVideo dataset, using the Faster RCNN detector with ResNet50 backbone. All the experimental settings are consistent with Section 4.2. L_i means the similarity loss of ith feature pyramid layer. For the ResNet50 backbone, L_1 is the loss of the feature map with the highest resolution (local) while L_4 is the loss of the feature map with the lowest resolution (global).

modified feature pyramid	mAP	AP_{50}	AP_{75}
	**24.3**	49.6	20.5
✓	23.7	49.4	19.8

Supplementary Table 2. Impact of modified feature pyramid and local patch-based losses for contrastive learning on downstream polyp detection performance. The experiments are conducted on the LDPolypVideo dataset, using the Faster RCNN detector with ResNet50 backbone. All the settings are consistent with Section 4.2.

Supplementary Figure

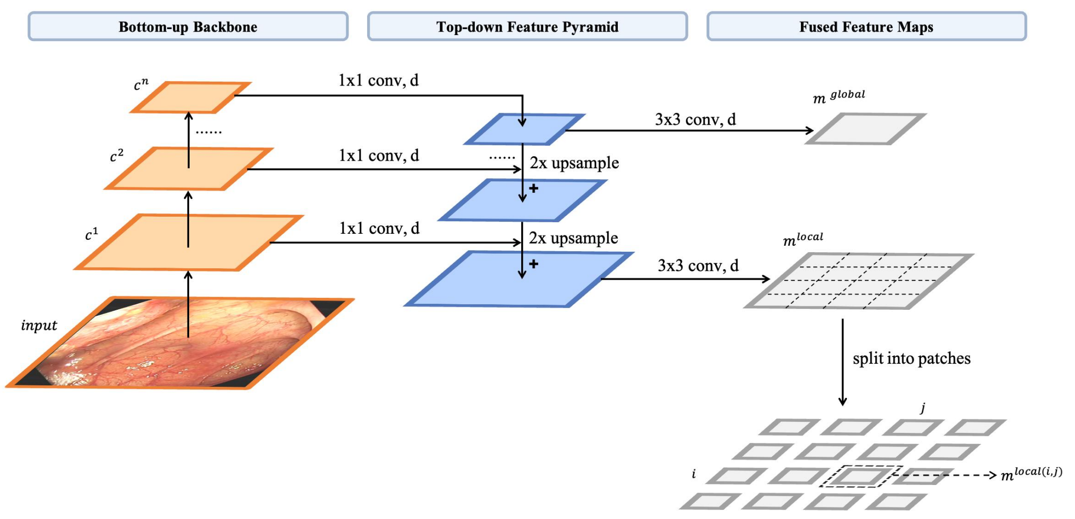

Supplementary Figure 1. A modified version of the feature pyramid to generate a single global feature map and dense local feature patches. The original single local feature map m^{local} is split into numerous local feature patches that have the same shape as the global feature map m^{global}. $m^{local(i,j)}$ denotes the local feature patch of the ith row and j^{th} column.
